# Whole-genome DNA methylation characteristics in pediatric precursor B cell acute lymphoblastic leukemia (BCP ALL)

**DOI:** 10.1371/journal.pone.0187422

**Published:** 2017-11-10

**Authors:** Radosław Chaber, Artur Gurgul, Grażyna Wróbel, Olga Haus, Anna Tomoń, Jerzy Kowalczyk, Tomasz Szmatoła, Igor Jasielczuk, Blanka Rybka, Renata Ryczan-Krawczyk, Ewa Duszeńko, Sylwia Stąpor, Krzysztof Ciebiera, Sylwia Paszek, Natalia Potocka, Christopher J. Arthur, Izabela Zawlik

**Affiliations:** 1 Institute of Nursing and Health Sciences, Faculty of Medicine, University of Rzeszow, Rzeszow, Poland; 2 National Research Institute of Animal Production, Laboratory of Genomics, Balice, Poland; 3 Department of Paediatric Bone Marrow Transplantation, Oncology and Hematology, Medical University of Wroclaw, Wroclaw, Poland; 4 Department of Clinical Genetics, Faculty of Medicine, Collegium Medicum in Bydgoszcz, Bydgoszcz, Nicolaus Copernicus University in Torun, Torun, Poland; 5 Department of Pediatric, Hematology, Oncology and Bone Marrow Transplantation, Medical University of Lublin, Lublin, Poland; 6 Department of Hematology, Medical University, Wroclaw, Poland; 7 Institute of Informatics, University of Warsaw, Warsaw, Poland; 8 Centre for Innovative Research in Medical and Natural Sciences, Laboratory of Molecular Biology, Faculty of Medicine, University of Rzeszow, Rzeszow, Poland; 9 School of Chemistry, University of Bristol, Bristol, United Kingdom; 10 Department of Genetics, Institution of Experimental and Clinical Medicine, University of Rzeszow, Rzeszow, Poland; German Cancer Research Center (DKFZ), GERMANY

## Abstract

In addition to genetic alterations, epigenetic abnormalities have been shown to underlie the pathogenesis of acute lymphoblastic leukemia (ALL)—the most common pediatric cancer. The purpose of this study was to characterize the whole genome DNA methylation profile in children with precursor B-cell ALL (BCP ALL) and to compare this profile with methylation observed in normal bone marrow samples. Additional efforts were made to correlate the observed methylation patterns with selected clinical features. We assessed DNA methylation from bone marrow samples obtained from 38 children with BCP ALL at the time of diagnosis along with 4 samples of normal bone marrow cells as controls using Infinium MethylationEPIC BeadChip Array. Patients were diagnosed and stratified into prognosis groups according to the BFM ALL IC 2009 protocol. The analysis of differentially methylated sites across the genome as well as promoter methylation profiles allowed clear separation of the leukemic and control samples into two clusters. 86.6% of the promoter-associated differentially methylated sites were hypermethylated in BCP ALL. Seven sites were found to correlate with the BFM ALL IC 2009 high risk group. Amongst these, one was located within the gene body of the *MBP* gene and another was within the promoter region- *PSMF1* gene. Differentially methylated sites that were significantly related with subsets of patients with *ETV6-RUNX1* fusion and hyperdiploidy. The analyzed translocations and change of genes’ sequence context does not affect methylation and methylation seems not to be a mechanism for the regulation of expression of the resulting fusion genes.

## Introduction

Acute lymphoblastic leukemia (ALL) is the most common pediatric cancer with 3–5 cases per 100,000 with a peak of incidence 2–5 years [1]. Most of these cases (80–85%) originate from precursor B cells, BCP ALL [2]. The initiation of BCP ALL (as with other ALL’s) is driven by genetic alterations including point mutations, chromosome amplifications or translocations which ultimately lead to abnormal expression of key genes responsible for cell proliferation and differentiation [3]. Indeed, leukemic cells in ~75% of patients contain some chromosome abnormalities. Some of these abnormalities have prognostic significance. For example, genetic changes with good prognosis include hyperdiploidy with greater than 50 chromosomes or translocation t(12;21) *ETV6-RUNX1 (TEL-AML1*) which are detected in about a quarter of cases of childhood BCP ALL [4]. Hypodiploidy with fewer than 44 chromosomes and chromosomal rearrangements including translocation t(9;22) *BCR-ABL1*, rearrangement of *MLL* at 11q23 to a diverse range of fusion partners and internal amplification of chromosome 21 (iAMP 21) are less common (1–6% of cases) but are prognostic of a significantly higher risk of relapse and poor outcome. There are also chromosomal abnormalities as t(1;19)(q23;p13) or *TCF3-PBX1* (formerly known as *E2A-PBX1)* (4% cases) with uncertain prognostic significance [4,5].

In addition to identifiable chromosomal abnormalities, many other recurring genetic alterations have been reported. The most prevalent submicroscopic alterations in pediatric ALL occur in *CDKN2A/B* (30–40%), *IKZF1* (15%), *PAX5 (*20%), and *ETV6* (10–15%). Most of these lesions have not been shown to impact prognosis. Mutations or deletions of *IKZF1*, located at 7p12, have, however, been shown to be independent predictors of outcome [5–8].

In addition to these genetic alterations, epigenetic abnormalities such as DNA methylation, post-translational histone protein modifications and interaction with non-coding RNA (miRNA or siRNA) have been shown to underlie the pathogenesis of ALL [9]. DNA methylation is a reversible process of attaching methyl residues to cytosine by the action of DNA methyltransferases—*DNMT1*, *DNMT3a*, *DNMT3b*. The methylation of cytosine’s located among CpG dinucleotides is significant for the control of gene expression. Such CpG sites may be dispersed amongst the whole genome but they frequently appear together in, so-called, CpG islands that are found within 70% of gene promoters [10] and are particularly common within the promoters of genes responsible for cell cycle control and cellular metabolism [11]. CpG islands are generally not methylated and methylation can inhibit gene expression both directly, through the prevention of transcription factors or RNA I polymerase binding, and indirectly by impacting on histone modification or chromatin remodeling proteins [12]. Aberrant methylation of CpG islands for the genes associated with DNA repair (*hMLH1*, *MGMT*), cell cycle control (*p16INK4A*, *p15INK4B*, *p14ARF*), apoptosis (*DAPK*) or detoxification (*GSTP1*) [11] can initiate carcinogenesis [11,13,14] including leukemogenesis [14–17].

The purpose of this study was to describe and characterize the whole genome DNA methylation profile in children with BCP ALL and to compare this with methylation profile obtained in normal bone marrow samples. The secondary aim was to correlate the methylation landscape with some biological and clinical features as characteristic of patients, cytogenetic aberrations and prognosis based on treatment protocol criteria.

## Material and methods

### Patients

Samples of bone marrow were obtained from 38 patients with pediatric BCP ALL at the time of diagnosis. The characteristics of patients are shown in Table 1. Ethics Committee approval was obtained from the Institutional Review Board of the Medical University of Lodz (number, RNN/226/11/KE). Informed consent has been obtained from parents/legal guardians of all the participating children. Various genetic aberrations were detected among most of the patients. The patients were stratified into prognostic groups according to the BFM ALL IC 2009 protocol [18]. This stratification is based on the initial clinical features including patient age, white-blood cells count at diagnosis, presence of specific genetic aberrations, the response to steroids at day 8, the cytomorphological response in bone marrow at day 15 and 33 and the minimal residual disease level at day 15.

**Table 1:** The characteristics of patients.

	No of pts.
**sex**	
male/female	21/17
**age**	
range [yrs]	1,5–17
median	5
risk group*	
high risk- HRG	5
intermediate risk- IRG	26
standard risk- SRG	7
the central nervous system involvement	3
relapse	1
death	2
prednisone poor responder	3
hematological remission at day 33	38
observation time	6–36 months
**cytogenetic aberrations**	
Hyperdiploidy (>50 chromosomes)	13
t(12;21) with fusion *ETV6-RUNX1*	7
t(1;19) with fusion *TCF3-PBX1*	3
hyper/hypo triploidy	3
IGH rearrangement	3
normal karyotype	2
others	7

* according ALL IC-BFM 2009 protocol [18]

Samples of normal bone marrow cells were examined as controls. These bone marrow samples were obtained during routine diagnostic procedures of other diseases (sideropenic anemia, localized Wilms tumor and localized lipofibromatosis) from 4 patients (2 male, 2 female, age 5–17 years). In each case, the bone marrow as well as microscopic examination of peripheral blood smears revealed no pathology.

### Samples and DNA methylation profiling

DNA was purified using QIAamp DNA Blood Mini Kit (QIAGEN), examined for integrity by agarose gel electrophoresis and quantified using Qubit 2.0 fluorimeter using a double stranded DNA (BR) assay (Thermo Fisher Scientific). About 500 ng of the sampled DNA was analyzed on a Infinium^®^ MethylationEPIC BeadChip (Illumina, San Diego, CA). The analysis comprised bisulfite conversion of DNA with EZ DNA Methylation^™^ Kit (Zymo Research) using modified thermal conditions (as recommended by the supplier). Purified converted DNA was then used as an input to Infinium HD Assay Methylation Protocol (Illumina). The hybridized and stained arrays were ultimately scanned using HiScanSQ system (Illumina). The Infinium MethylationEPIC BeadChip used enabled the analysis of more than 850,000 methylation sites per sample which cover the broad content categories, including sites: within known CpG islands, outside of CpG islands, Non-CpG methylated sites identified in human stem cells (CHH sites), differentially methylated (DM) sites identified in tumor versus normal (multiple forms of cancer) and across several tissue types, FANTOM5 enhancers, ENCODE open chromatin and enhancers, DNase hypersensitivity sites and miRNA promoter regions. Moreover, the array content covers >90% of content contained on the previous Illumina HumanMethylation450 BeadChip.

### Data quality control and analysis

The raw intensity data were assessed for quality using BeadArray Controls Reporter (Illumina). Next, the obtained IDAT files were analyzed using the Chip Analysis Methylation Pipeline (ChAMP) [19] for EPIC array data. During initial data handling, probes with p-value <0.01 and with fewer than 3 beads in at least 5% of samples were excluded. Additionally, non-CpG probes, SNP-related probes, multi-hit probes and probes located in chromosome X and Y were also removed. The beta values for 800,619 probes per sample were then calculated and checked for quality by evaluation of a beta multidimensional salting (MDS) plot and a density plot across the study groups. The beta value is defined as a proportion of DNA methylation at a particular CpG site (also called the methylation beta-value (β)) which is ascertained by taking the ratio of the methylated (C) to unmethylated (T) signals, using the formula: β = intensity of the methylated signal/(intensity of the unmethylated signal + intensity of the methylated signal + 100). A β-value of 0 represents an unmethylated CpG site and a β-value approaching 1 represents a fully methylated CpG site.

The obtained beta values were then normalized using the BMIQ method [20] and the singular value decomposition method (SVD) implemented by Teschendorff *et al*. (2009) [21] was used to identify the most significant causes of variation, including technical variation (batch effects like: slide, array or sample well). Batch effects (if detected for separate comparisons) were removed using the ComBat algorithm which uses an empirical Bayes method designed to correct data for technical variation [22].

Differential probes methylation between groups was calculated using the champ.DMP() function which uses the Limma package [23] to calculate the p-value for differential methylation using a linear model. The DMP detecting t-test p-values were corrected for multiple testing using the Benjamin-Hochberg procedure [24].

Differential methylation analysis was performed in three setups: to identify the general differences between all BCP ALL patients and the controls, second to identify sites/regions differentially methylated between *SRG/IRG* and *HRG* patients and third detecting methylation sites associated with recurrent genetic abnormalities. Additional pairwise comparisons have been made within BCP ALL patients group to detect sites which may be associated with confounding factors such as age (≤6 vs. >6 years of age) or gender (males vs. females). Differences in the distribution of CpG sites across different genomic classes (gene bodies, IGR, promoters, CpG islands etc.) were evaluated using Chi-square test.

### Functional genes annotation and analysis

The genes associated with specific differentially methylated (DM) sites were analyzed in terms of molecular functions, biological presses, cellular components, pathways and phenotypes using WebGestalt (WEB-based GEne SeT AnaLysis) toolkit, exploiting an information obtained from GO, KEGG, WikiPathways, Human Phenotype Ontology and PharmGKB databases. Enrichment analysis was performed with respect to all known human genes (genome), identifying enriched categories with corrected p-value (according to Benjamin-Hochberg procedure) lower than 0.01 and requiring at least 4 genes per enriched category.

## Results

### General assay performance and CpG statistics

The general assay performance was validated using control probes and no issues were found with the samples. After initial filtration, the beta values of 800,619 probes were normalized and batch effect was identified (slide, array and sample well) within the data by evaluation of components of variation. After normalization for technical variation, SVD indicated that the only source of variation amongst the sample groups was inter-group differentiation. Among the valid probes, representing individual CpG sites, 295,458 were located within genes (genes body), 228,710 in intergenic regions (IGR) and 67,841 in genes’ 5’UTR. Promoter associated sites, defined as: TSS200 (0–200 nucleotides (nt) upstream of the transcription start site) and TSS1500 (200–1500 nt upstream of TSS) were represented by 98,881 and 60,483 CpGs, respectively. 150,080 analyzed probes were located within known CpG islands, 142,693 in “shores” (up to 2000 bp from island) and 453,065 probes were located outside CpG islands (open sea).

### Genome wide differential methylation analysis between BCP ALL and control

**Fig 1:**
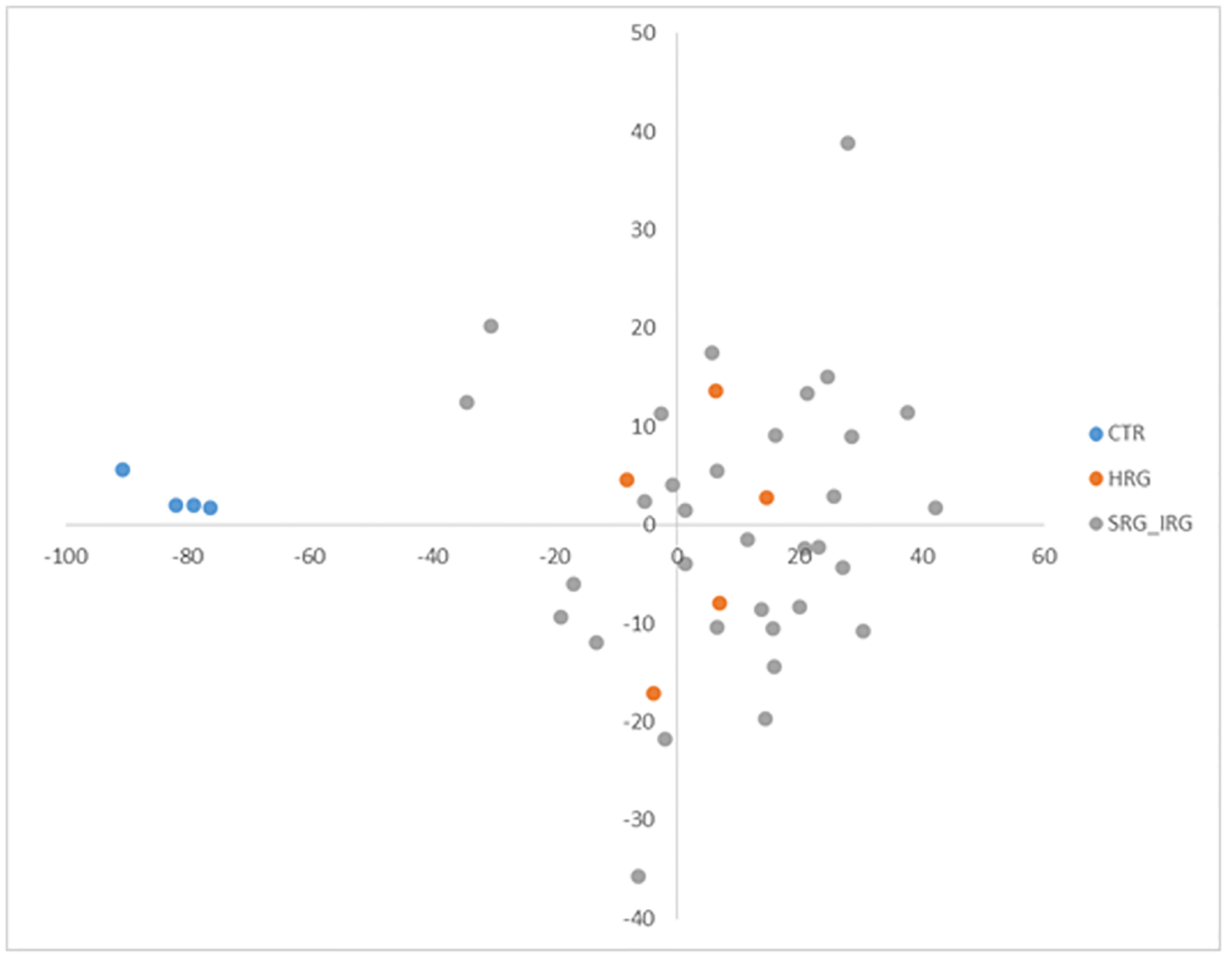
PCA based on 118,871 sites differentially methylated between BCP ALL and control samples.

**Fig 2:**
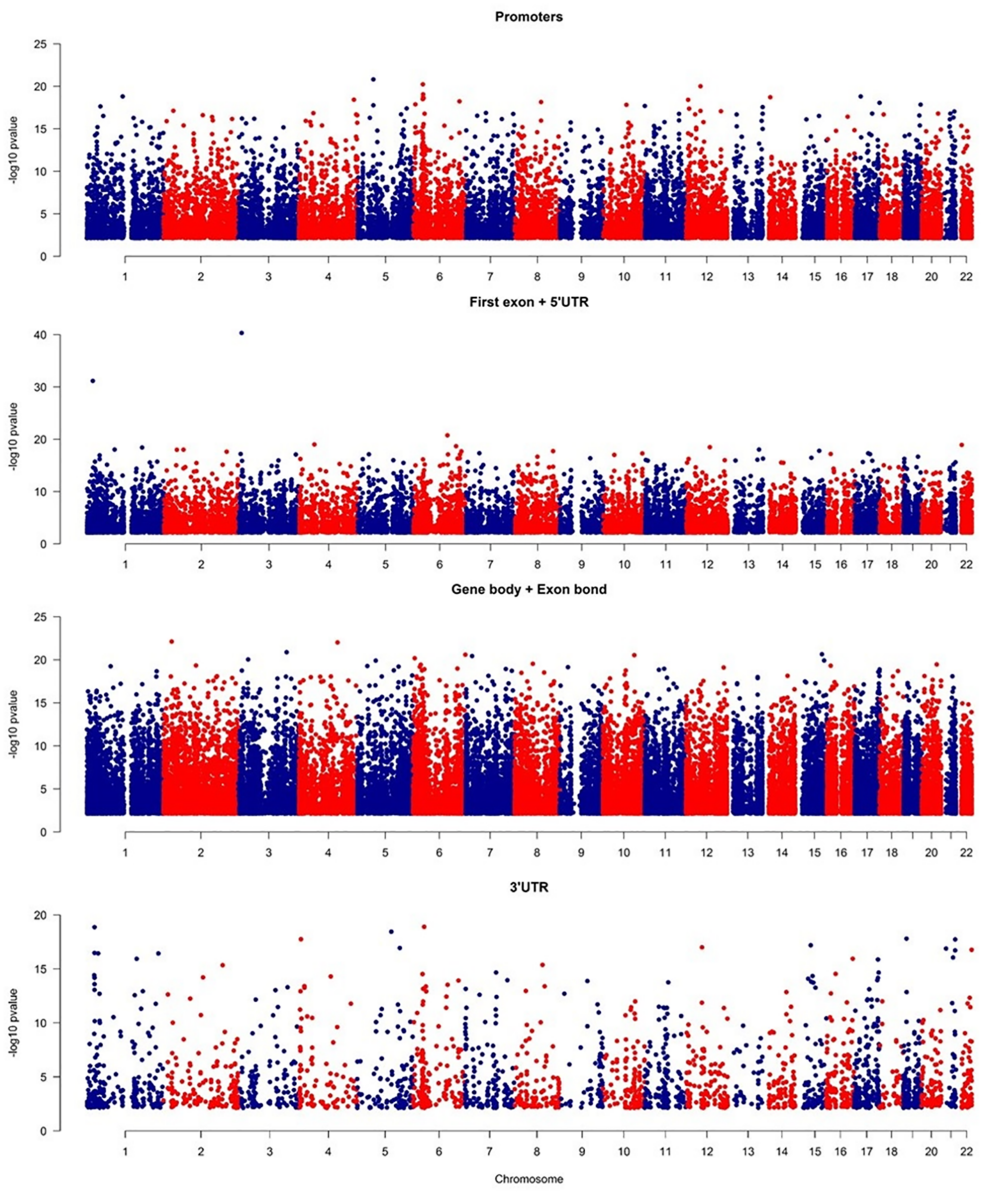
Genomic distribution of CpGs with significant differences in methylation level between BCP ALL and control in gene context.

The DNA methylation status of 800,619 CpG loci distributed across the genome was interrogated in 38 pediatric BCP ALL patients and four non-leukemic control samples. A common set of 118,871 probes, mapped to 17,885 unique genes (promoters and gene bodies) or intergenic regions, were found to be differentially methylated (adjP<0.05; DM) in the patient’s relative to controls, suggesting the presence of distinct BCP ALL-associated DNA methylation signature (S1 Appendix). The absolute Δβ value for separate DM probes (calculated as an absolute difference between averaged β-values for separate groups) ranged from 0.009 to 0.730 with a mean of 0.216. Applying principal component analysis (PCA) to the differentially methylated site data shows clear separation of leukemic and control methylation profiles, with a higher level of profile variation observed amongst the BCP ALL samples (Fig 1). DM sites are uniformly distributed across the genome (Fig 2 as a Manhattan plot of -log_10_p-values for differentially methylated probes).

**Fig 3:**
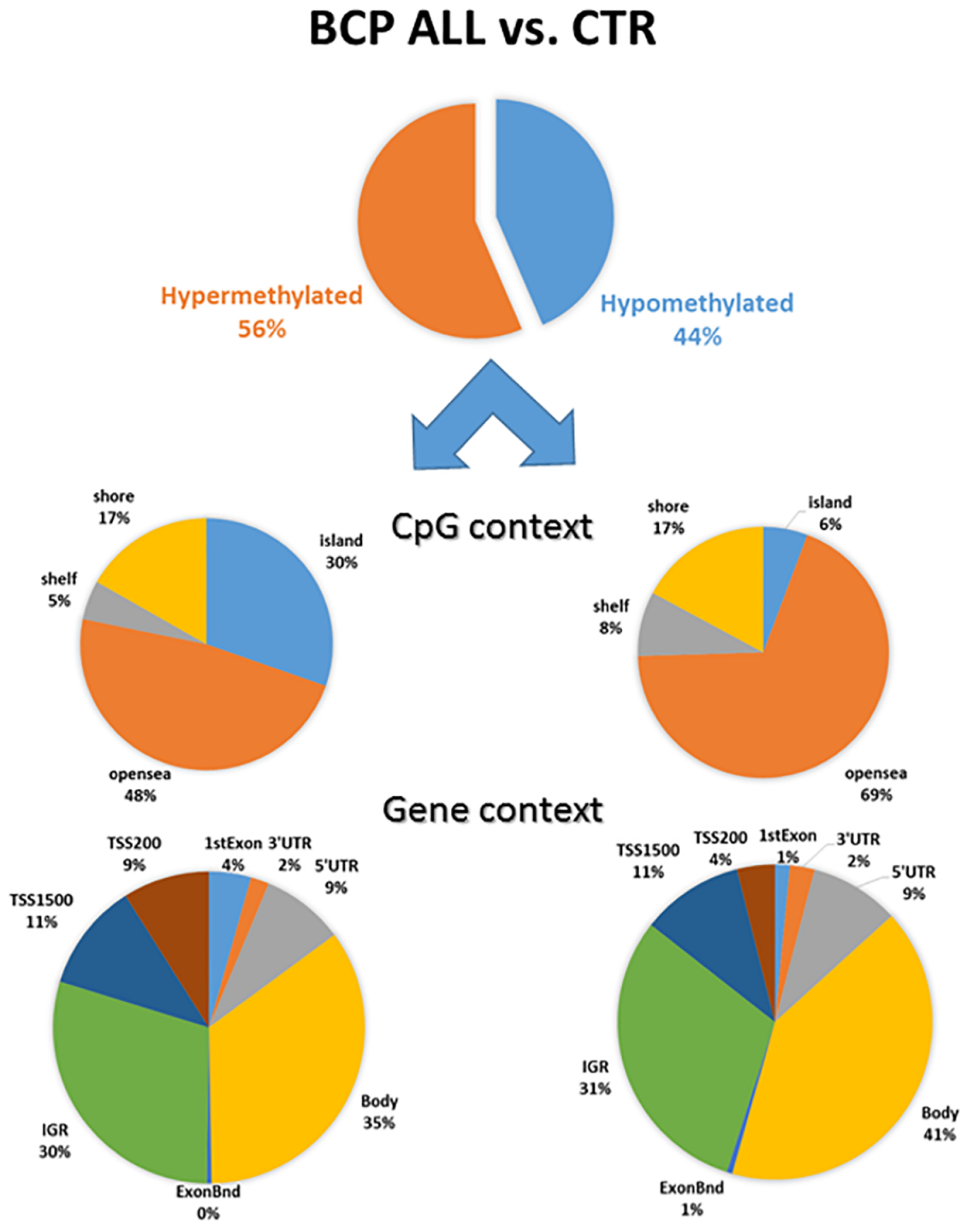
Distribution of hyper- and hypomethylated sites differentially methylated between leukemic and control samples.

Of the 118,871 sites differentially methylated between leukemic and control samples, 67,115 (56%) were hypermethylated in BCP ALL samples and 51,756 (44%) were hypomethylated. The annotation analysis and comparison of hypo- and hypermethylated CpG features showed that genome-wide hypermethylation in leukemic DNA is more commonly associated with CpG islands and regions in close vicinity of transcription start site (TSS200 and 1st exon), whereas hypomethylation is more common in gene bodies and regions outside CpG islands (open-sea) (Fig 3, Table 2). The differences in distribution of hyper- and hypomethylated sites among separate genomic regions were statistically significant for all CpG context classes (as shown by Chi-square test; p<0.001).

**Table 2:** Genomic distribution of probes differentially methylated between leukemic and control samples with respect to CpG island and gene context.

Centex	Differentially methylated sites between BCP ALL and control		Differentially methylated between BCP ALL and control with subdivision into hyper- and hypo-methylated sites			
Region	No of probes	% of probes	No of Hyper- in BCP ALL	% of Hyper-	No of Hypo- in BCP ALL	% of Hypo-
Gene context						
1stExon	3719	3.1	2929	4.4	790	1.5
3'UTR	2591	2.2	1280	1.9	1311	2.5
5'UTR	10536	8.9	5779	8.6	4757	9.2
Body	44674	37.6	23388	34.8	21286	41.1
ExonBnd	635	0.5	330	0.5	305	0.6
IGR	35673	30.0	19831	29.5	15842	30.6
TSS1500	13004	10.9	7552	11.3	5452	10.5
TSS200	8039	6.8	6026	9.0	2013	3.9
CpG island context						
island	23323	19.6	20369	30.3	2954	5.7
opensea	67759	57.0	32128	47.9	35631	68.8
shelf	7665	6.4	3362	5.0	4303	8.3
shore	20124	16.9	11256	16.8	8868	17.1
Gene/island context						
1stExon-island	2473	2.1	2247	3.3	226	0.4
1stExon-opensea	732	0.6	344	0.5	388	0.7
1stExon-shelf	49	0.0	25	0.0	24	0.0
1stExon-shore	465	0.4	313	0.5	152	0.3
3'UTR-island	257	0.2	183	0.3	74	0.1
3'UTR-opensea	1593	1.3	780	1.2	813	1.6
3'UTR-shelf	252	0.2	86	0.1	166	0.3
3'UTR-shore	489	0.4	231	0.3	258	0.5
5'UTR-island	2376	2.0	2063	3.1	313	0.6
5'UTR-opensea	5433	4.6	2459	3.7	2974	5.7
5'UTR-shelf	883	0.7	355	0.5	528	1.0
5'UTR-shore	1844	1.6	902	1.3	942	1.8
Body-island	5650	4.8	4718	7.0	932	1.8
Body-opensea	29807	25.1	14168	21.1	15639	30.2
Body-shelf	3361	2.8	1502	2.2	1859	3.6
Body-shore	5856	4.9	3000	4.5	2856	5.5
ExonBnd-island	19	0.0	13	0.0	6	0.0
ExonBnd-opensea	541	0.5	279	0.4	262	0.5
ExonBnd-shelf	33	0.0	19	0.0	14	0.0
ExonBnd-shore	42	0.0	19	0.0	23	0.0
IGR-island	5029	4.2	4621	6.9	408	0.8
IGR-opensea	24351	20.5	11634	17.3	12717	24.6
IGR-shelf	2548	2.1	1165	1.7	1383	2.7
IGR-shore	3745	3.2	2411	3.6	1334	2.6
TSS1500-island	3052	2.6	2605	3.9	447	0.9
TSS1500-opensea	3482	2.9	1531	2.3	1951	3.8
TSS1500-shelf	351	0.3	136	0.2	215	0.4
TSS1500-shore	6119	5.1	3280	4.9	2839	5.5
TSS200-island	4467	3.8	3919	5.8	548	1.1
TSS200-opensea	1820	1.5	933	1.4	887	1.7
TSS200-shelf	188	0.2	74	0.1	114	0.2
TSS200-shore	1564	1.3	1100	1.6	464	0.9

Probes distribution in separate categories differed significantly across whole table with p<0.001

### Differential methylation analysis of genes’ promoters in BCP ALL and control samples

To characterize changes in methylation profile of genes’ promoters accompanying leukemia progression, we focused on a differentially methylated CpGs (BCP ALL vs. CTR) located in regions upstream from transcription start sites (TSS200, TSS1500), within CpG islands or in their direct vicinity (island shore; ±2000 bp). DM sites were additionally filtered to remove those with absolute Δβ value between groups lower than 0.3. This resulted in selection of 5,465 DM CpGs of which 2,611 (47.8%) were in a range of 200 bp from TSS and the remaining 2,854 were in regions from 200 to 1500 bp from TSS. The majority of the sites (3,451; 63.1%) were positioned within CpG island cores and the remaining were in regions upstream or downstream of the islands (island shores; 2,014 CpGs). The average absolute Δβ value for the promoters-associated sites after filtration was 0.399 (±0.076).

The vast majority of the promoter-associated DM sites (4733; 86.6%) were hypermethylated in leukemia when compared to healthy control. Hypermethylated CpGs were located both in TSS200 (53.4%) and TSS1500 (46.6%) promoter regions and were mainly found within CpG islands (72.5%), whereas hypomethylated sites occurred predominantly in regions positioned further away from TSS (TSS1500; 88.7%) and outside of CpG islands (island shores; 97.5%).

**Fig 4:**
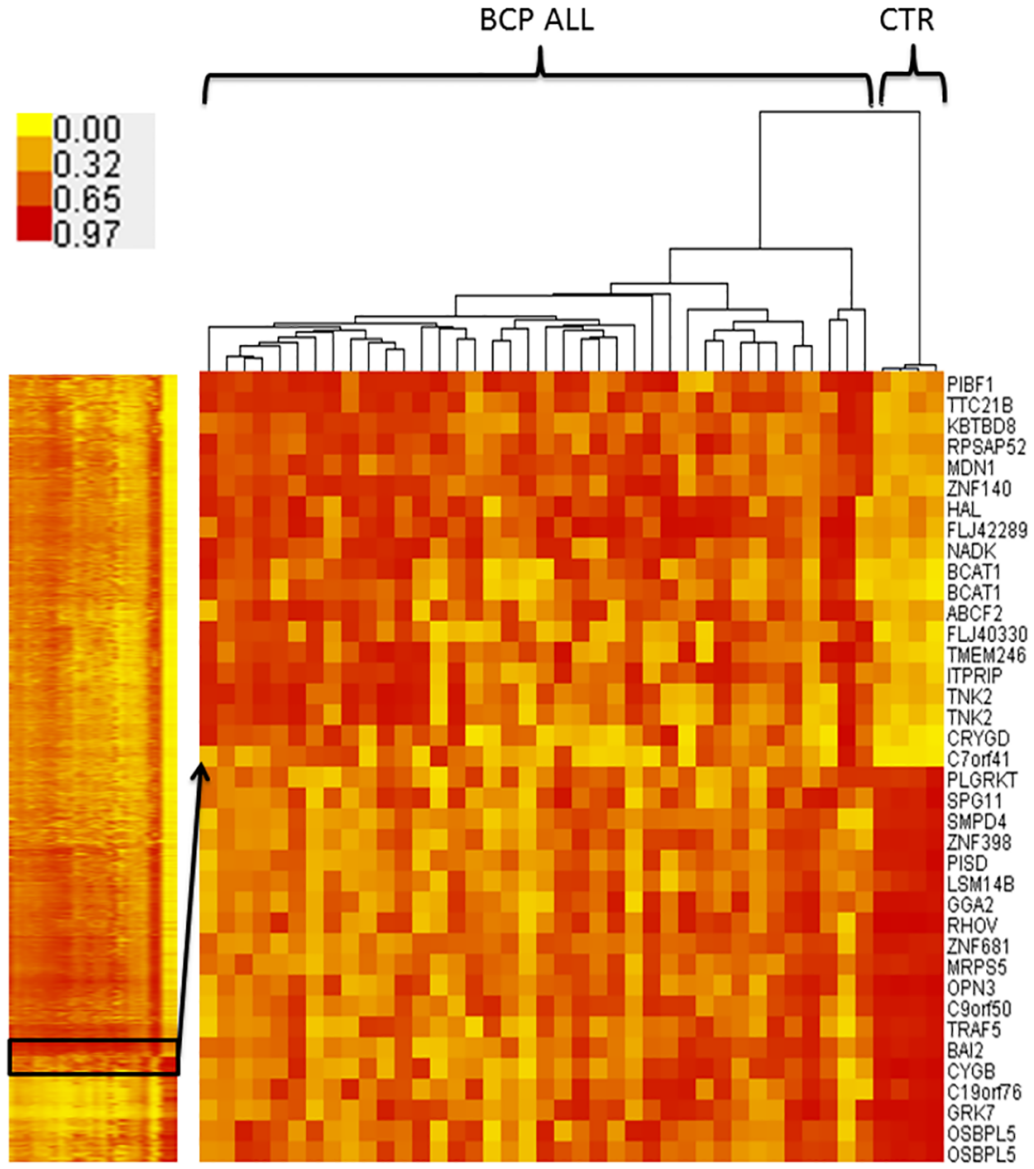
Unsupervised hierarchical clustering of promoter regions-associated methylation profiles in leukemic and control samples.

Unsupervised hierarchical clustering (based on Euclidean distance) of the promoter methylation profiles showed clear separation of the leukemic and non-leukemic samples into two visible clusters (Fig 4) proving the existence of distinct methylation patterns amongst genomic promoters. Some variation was also observed within BCP ALL group with visible separation of three leukemic samples.

### Identification of a leukemia-characteristic methylation profile

**Fig 5:**
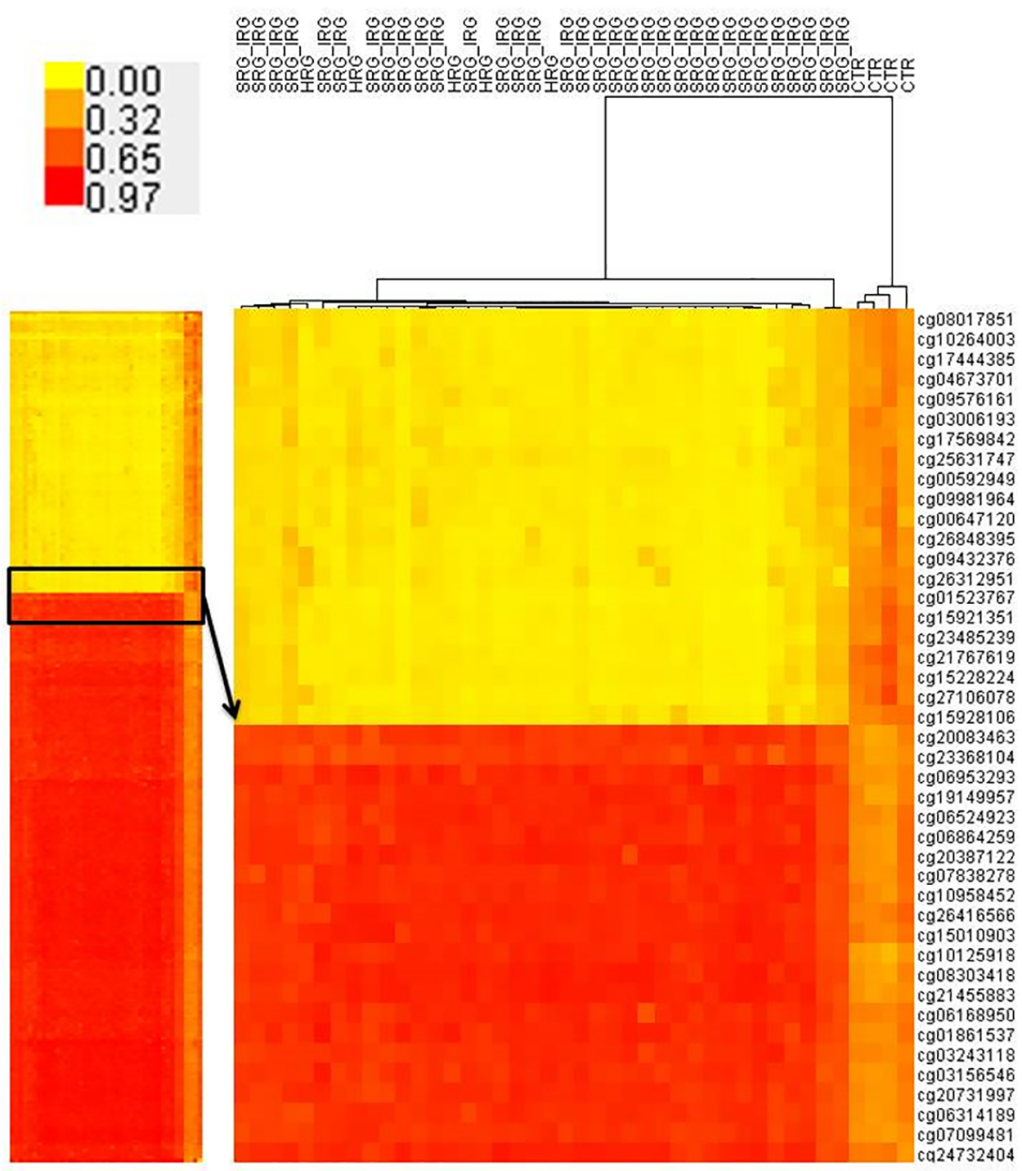
Unsupervised hierarchical clustering of methylation profiles with probes selected to minimize variation among leukemic samples.

**Fig 6:**
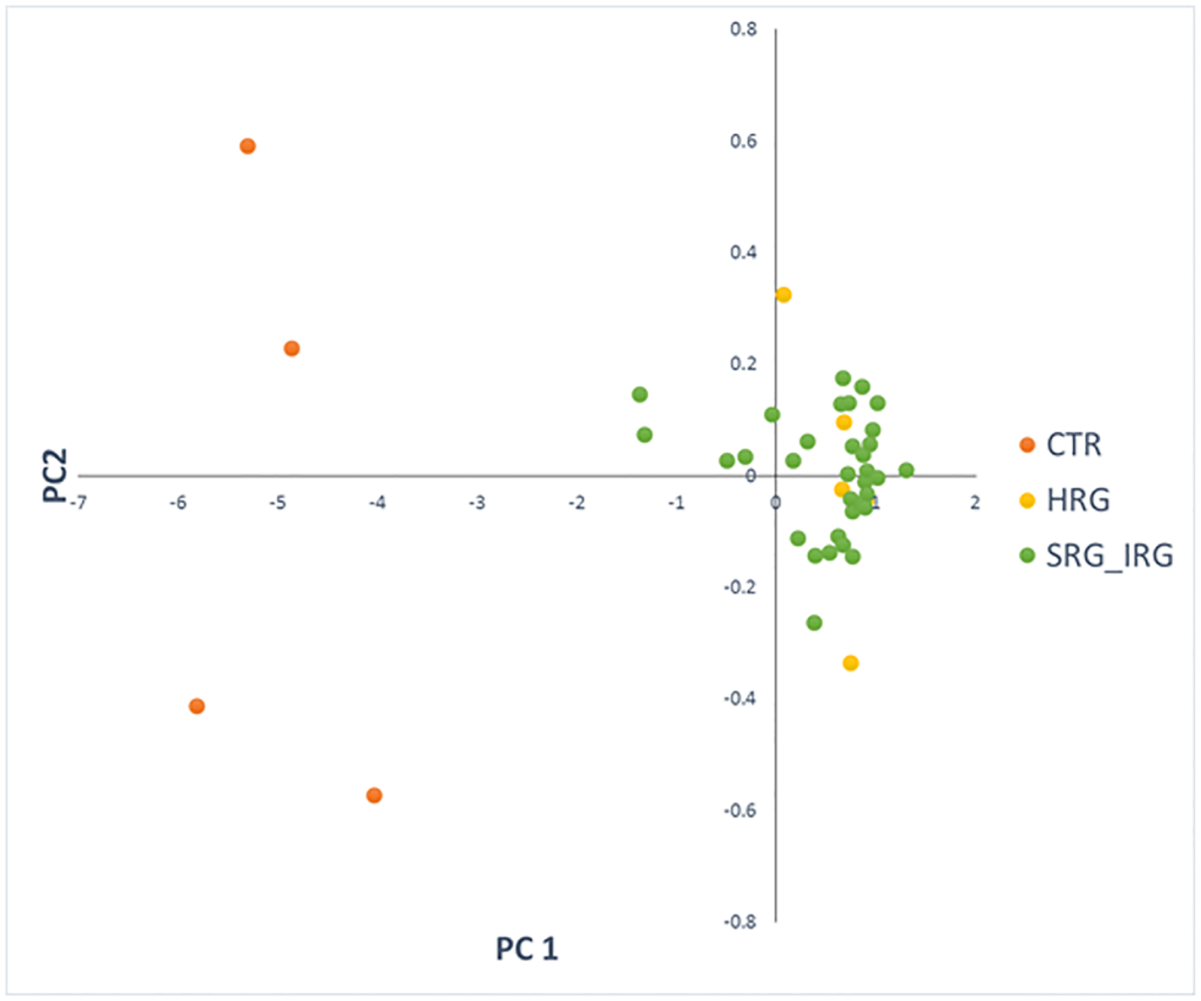
PCA based on filtered probes set minimizing the variation of methylation profiles in leukemic samples.

To find the most uniform and distinct from physiological methylation signature for leukemia, additional filtering criteria of all probes differentially methylated between BCP ALL and control were applied. The procedure aimed to identify CpGs with the lowest methylation level variation amongst BCP ALL samples (beta values standard deviation <0.05 within BCP ALL group) and with the largest difference in methylation level with respect to the control (delta beta absolute value >0.3). This filtration step allowed a set of 265 DM CpGs to be identified, with the most uniform and distinct from the methylation profile of the non-leukemic samples. As expected, unsupervised hierarchical clustering based on these probes enabled clear separation of the leukemic and non-leukemic methylation profiles and demonstrated high profile uniformity across BCP ALL samples (Fig 5) with minor discrepancies found for two leukemic samples diagnosed as BCP ALL with prodromal, preleukemic phase. This observation was also confirmed by PCA which showed that with the use of filtered probes set, low and lower than in control group level of methylation profile variation was observed in BCP ALL (Fig 6).

The potentially leukemia-characteristic DM sites were distributed on all 22 autosomes and were predominately located in gene bodies (48.7%) or intergenic regions (26.8%) and most (83.0%) were distributed in regions outside of CpG islands (open sea). The hypermethylated sites (n = 177; 66.8%) showed similar regional distribution as hypomethylated with slightly increased share of probes located within gene bodies. The detailed statistics on the selected CpG are presented in S2 Appendix.

### CpG associated with leukemia risk (SRG/IRG vs. HRG)

To find CpG sites which methylation level is associated with leukemia risk, the BCP ALL cases were stratified into two groups, encompassing *HRG* and combined *SRG/IRG* patients according to the BFM ALL IC 2009 protocol [18]. Statistical analysis showed, that of the 800,619 CpG analyzed sites, only 14 differed in methylation level between the groups. Most of these sites (n = 10; 71.4%) were hypomethylated in *HRG*. The average absolute Δβ for these sites was low with a mean of 0.232 (±0.058). Eight of the differentially methylated CpGs were located in intergenic regions and only two were found within promoter regions (TSS1500). Another three DM CpG were positioned within gene bodies. The list of DM sites between *HRG* and *SRG/IRG* is presented in S3 Appendix.

**Fig 7:**
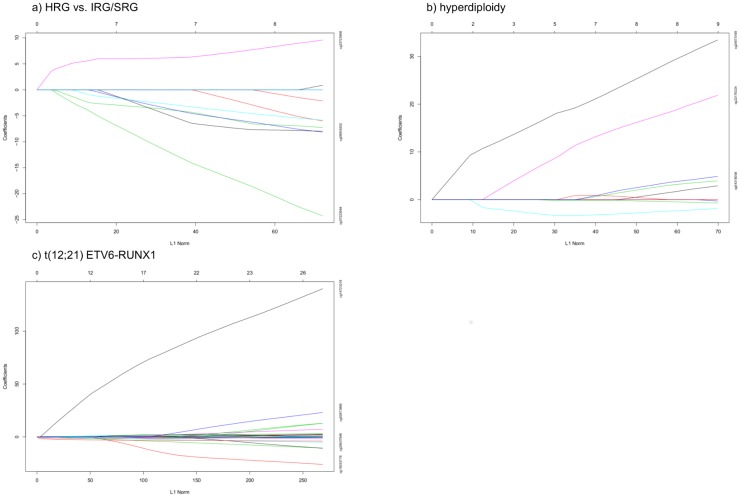
Results of Ridge Regression of a high risk of a patient in the analyzed data set. Each curve corresponds to a variable. It shows how much this variable contributes to the prediction of high risk of patient (a), hyperdiploidy (b) and t(12;21) ETV6-RUNX1 (c) aberrations in analyzed data set. Numbers on the left are coefficients and numbers on top are total count of variables selected for an L1 Norm (statistical parameter) shown on the bottom.

The most important CpG sites for prognosis were identified using a lasso penalized logistic regression [25]. This identified seven sites which are correlated with prognosis. Six of these relate to high-risk group stratification with poorer prognosis when they were hypomethylated and one if hypermethylated (Fig 7a). Among these selected CpG two were located within genes body or were promoter region-related (corresponding to *MBP* and *PSMF1* gene) and five are found in intergenic regions.

### Age and gender-related methylation changes in leukemia patients

**Fig 8:**
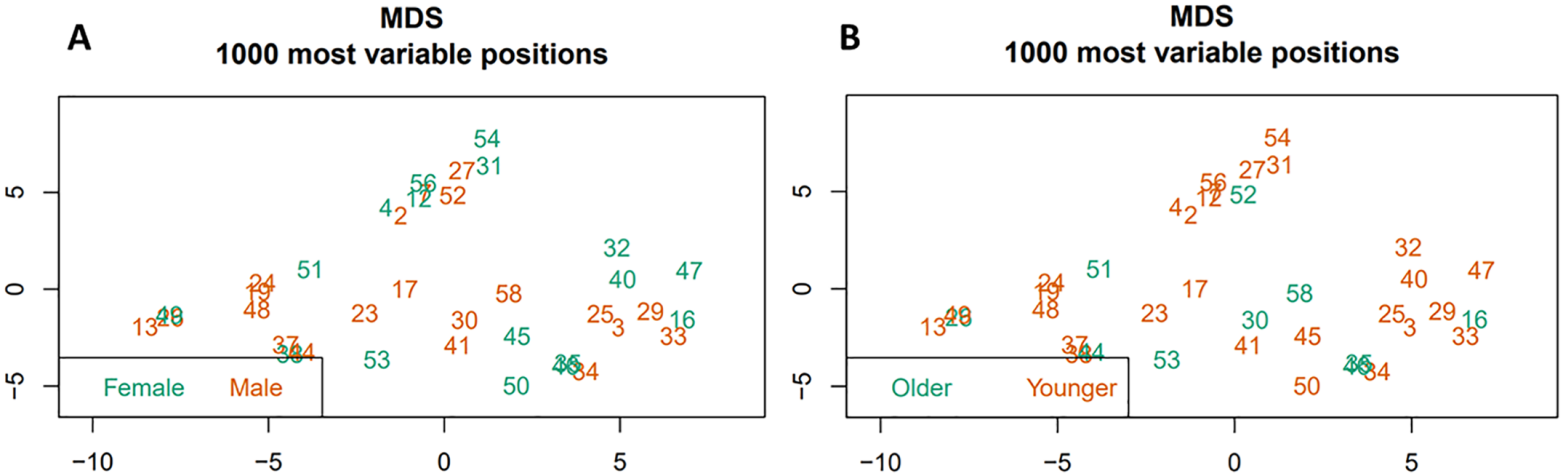
Multidimensional scaling analysis based on 1000 the most variable sites with respect to: A—gender, B—age.

Age and gender-related changes in CpG methylation in leukemia patients were identified in two separate comparisons by the stratification of BCP ALL group according to sex and age (≤6 and >6 years of age). A MDS analysis based on 1000 the most variable sites did not show clear stratification of methylation profiles according to these two conditions (Fig 8).

The identification of DM probes between separate subgroups allowed 113 sites (connected with 50 different genes) that differ in methylation depending on sex and 66 (36 genes) connected with age. Of the gender-related sites, 77 (68.1%) were hypermethylated in females with low level of methylation differences between sexes, expressed by a mean absolute Δβ of 0.143. The age-related changes were mostly linked with hypermethylation of CpGs, with almost 85% of probes showing higher methylation levels in older patients. The extent of age related methylation was higher than this observed for sex, with mean absolute delta beta of 0.329 (±0.073). Only three of the sex-related DM sites and seven of the age-related sites have been found among previously detected 118,871 CpGs differing in methylation levels between BCP ALL cases and controls, suggesting that age and sex are not important confounding factors for the methylation profile differentiation of pediatric leukemia.

### Changes in methylation patterns associated with recurrent genetic abnormalities in childhood ALL

To find methylation profiles distinctive for genetic BCP ALL subtypes, patients were stratified into groups with *ETV6-RUNX1* (n = 7), *TCF3-PBX1* (n = 3), *IGH* (n = 3), hyperdiploidy (n = 13) and triploidy (n = 3) cytogenetic variants. Differentially methylated sites were identified in pairwise comparisons of specific genetic subtype with all other patients with known cytogenetic status, including eight patients without major chromosomal aberrations. Differential methylation analysis identified from 117 to 6,977 sites for the separate genetic subtypes. Of these, only a few (up to 67) overlapped between the separate groups. All analyzed subtypes were associated with hypomethylation with respect to the remaining patients with the highest proportion of hypomethylated sites found in *IGH*, hyperdiploidy and triploidy aberration carriers (from 72 to 77%) (Table 3). The distribution of DM sites across the genome was generally similar for separate subtypes, however, some evidence for an increased number of DM sites in the TSS1500 promoter regions in *IGH* carries have been found. The highest differences in methylation level compared to remaining BCP ALL cases, measured as average Δβ (0.384) were observed for *TCF3-PBX1* genetic subtype (Table 3). The DM sites were related to 74 to 2963 unique genes depending on genetic abnormality and total number of detected DM sites. These genes are associated with a broad range of KEGG pathways, of which the top ten are presented in Table 4. The list of DM probes along with their annotation and associated genes is presented in S4 Appendix.

**Table 3:** Statistic of sites differentially methylated between specific genetic subtypes of leukemia and remaining BCP ALL patients with known cytogenetic status.

	Genetic subtype				
	*ETV6-RUNX1*	*TCF3-PBX1*	*IGH*	Hyperdiploidy	Triploidy
	Number of DM sites				
All DM sites	5207	6977	117	4401	347
	Percentage of hyper-/hypomethylated sites				
Hyper-	40.3	36.2	23.1	27.5	24.8
Hypo-	59.7	63.8	76.9	72.5	75.2
	Localization in gene context (% of all sites)				
1stExon	3.5	1.4	0.9	1.5	3.2
3'UTR	2.0	2.9	5.1	2.1	0.9
5'UTR	12.5	8.6	6.0	7.3	11.0
Body	36.5	44.3	28.2	39.6	33.7
ExonBnd	0.4	0.8	3.4	0.7	1.2
IGR	26.9	28.6	33.3	36.3	29.4
TSS1500	10.4	8.9	19.7	8.9	11.0
TSS200	7.7	4.5	3.4	3.7	9.8
	Localization in CpG island context (% of all sites)				
island	21.6	9.6	7.7	7.1	16.4
opensea	55.7	66.2	65.0	75.6	60.5
shelf	7.0	8.1	5.1	5.6	7.2
shore	15.7	16.1	22.2	11.7	15.9
	Average difference in methylation				
all	0.272	0.384	0.208	0.243	0.171
hyper	0.252	0.37	0.209	0.252	0.144
hypo	-0.285	-0.392	-0.207	-0.239	-0.179
	Number of associated genes				
Unique genes	2065	2963	74	1836	205

**Table 4:** Top ten KEGG pathways connected with genes containing sites differentially methylated between specific genetic subtype and remaining BCP ALL patients.

ID	Name	Number of Genes	FDR
*ETV6-RUNX1*			
hsa05200	Pathways in cancer	83	2.24E-09
hsa04724	Glutamatergic synapse	31	0.0000131
hsa04713	Circadian entrainment	27	0.0000284
hsa04728	Dopaminergic synapse	32	0.0000489
hsa04723	Retrograde endocannabinoid signaling	27	0.000052
hsa04360	Axon guidance	38	0.000118
hsa04015	Rap1 signaling pathway	42	0.000268
hsa04925	Aldosterone synthesis and secretion	22	0.000282
hsa04921	Oxytocin signaling pathway	34	0.000282
hsa04659	Th17 cell differentiation	26	0.000282
*TCF3-PBX1*			
hsa04071	Sphingolipid signaling pathway	38	0.000254
hsa04070	Phosphatidylinositol signaling system	32	0.000391
hsa04360	Axon guidance	48	0.000391
hsa05200	Pathways in cancer	89	0.000391
hsa04144	Endocytosis	62	0.00125
hsa04921	Oxytocin signaling pathway	42	0.00168
hsa04072	Phospholipase D signaling pathway	39	0.00168
hsa04520	Adherens junction	24	0.0021
hsa05221	Acute myeloid leukemia	20	0.0021
hsa04066	HIF-1 signaling pathway	30	0.0021
*IGH*			
hsa04730	Long-term depression	2	1
hsa04920	Adipocytokine signaling pathway	2	1
hsa00562	Inositol phosphate metabolism	2	1
hsa05146	Amoebiasis	2	1
hsa04916	Melanogenesis	2	1
hsa04080	Neuroactive ligand-receptor interaction	3	1
hsa04611	Platelet activation	2	1
hsa04152	AMPK signaling pathway	2	1
hsa04120	Ubiquitin mediated proteolysis	2	1
hsa04310	Wnt signaling pathway	2	1
Hiperdploidy			
hsa05200	Pathways in cancer	65	0.00000743
hsa01521	EGFR tyrosine kinase inhibitor resistance	22	0.0000447
hsa04360	Axon guidance	35	0.0000607
hsa04728	Dopaminergic synapse	28	0.000106
hsa04015	Rap1 signaling pathway	38	0.000155
hsa04723	Retrograde endocannabinoid signaling	23	0.00023
hsa04151	PI3K-Akt signaling pathway	52	0.000262
hsa04261	Adrenergic signaling in cardiomyocytes	29	0.000294
hsa04724	Glutamatergic synapse	24	0.000403
hsa04713	Circadian entrainment	21	0.000655
Triploidy			
hsa05213	Endometrial cancer	4	0.184
hsa05223	Non-small cell lung cancer	4	0.184
hsa05221	Acute myeloid leukemia	4	0.184
hsa04916	Melanogenesis	5	0.184
hsa04664	Fc epsilon RI signaling pathway	4	0.207
hsa04724	Glutamatergic synapse	5	0.207
hsa05100	Bacterial invasion of epithelial cells	4	0.255
hsa01521	EGFR tyrosine kinase inhibitor resistance	4	0.255
hsa04024	cAMP signaling pathway	6	0.274
hsa04072	Phospholipase D signaling pathway	5	0.274

**Fig 9:**
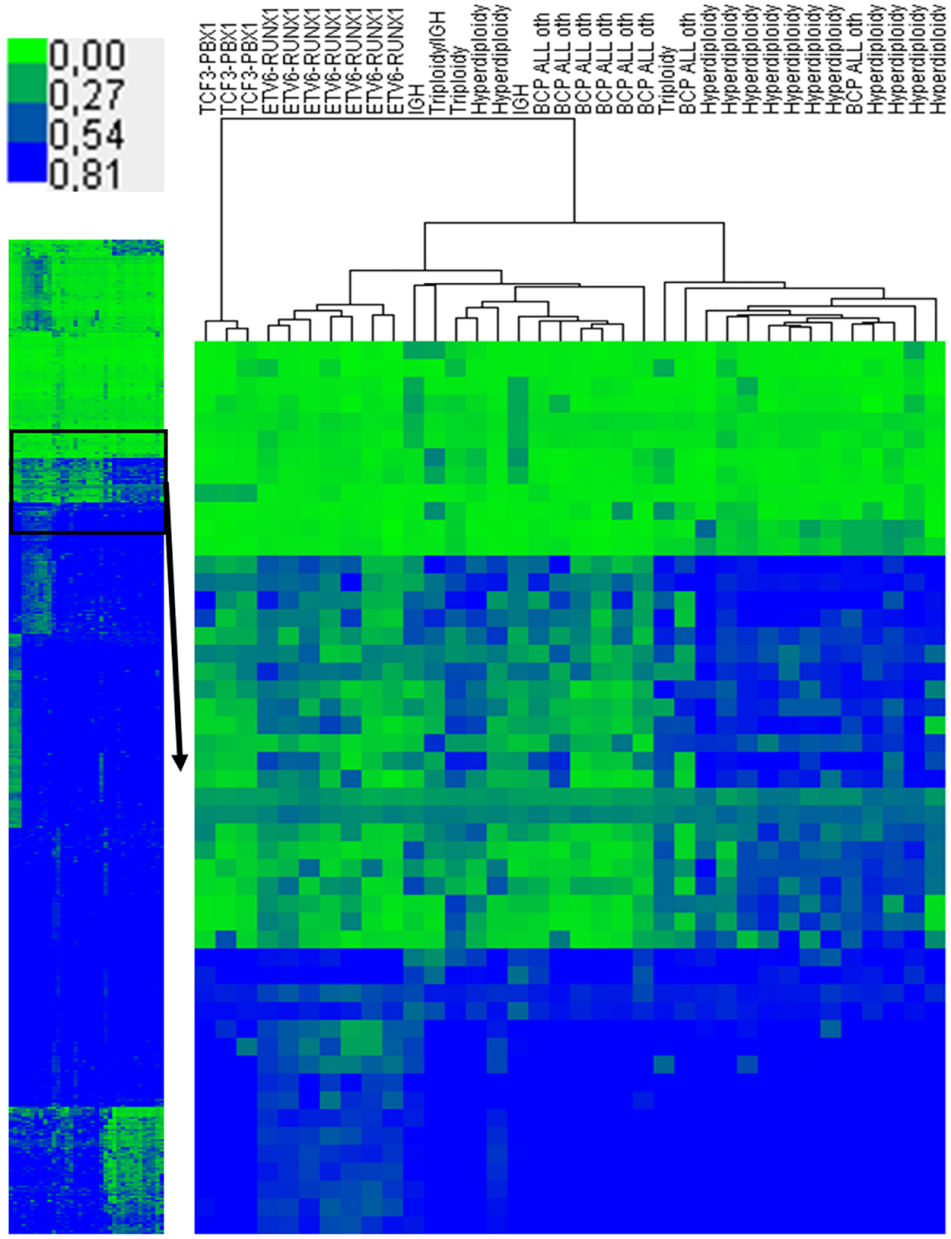
The unsupervised hierarchical clustering of the methylation level of a panel of 500 probes with the largest differences in methylation level between different leukemia genetic subtypes.

**Fig 10:**
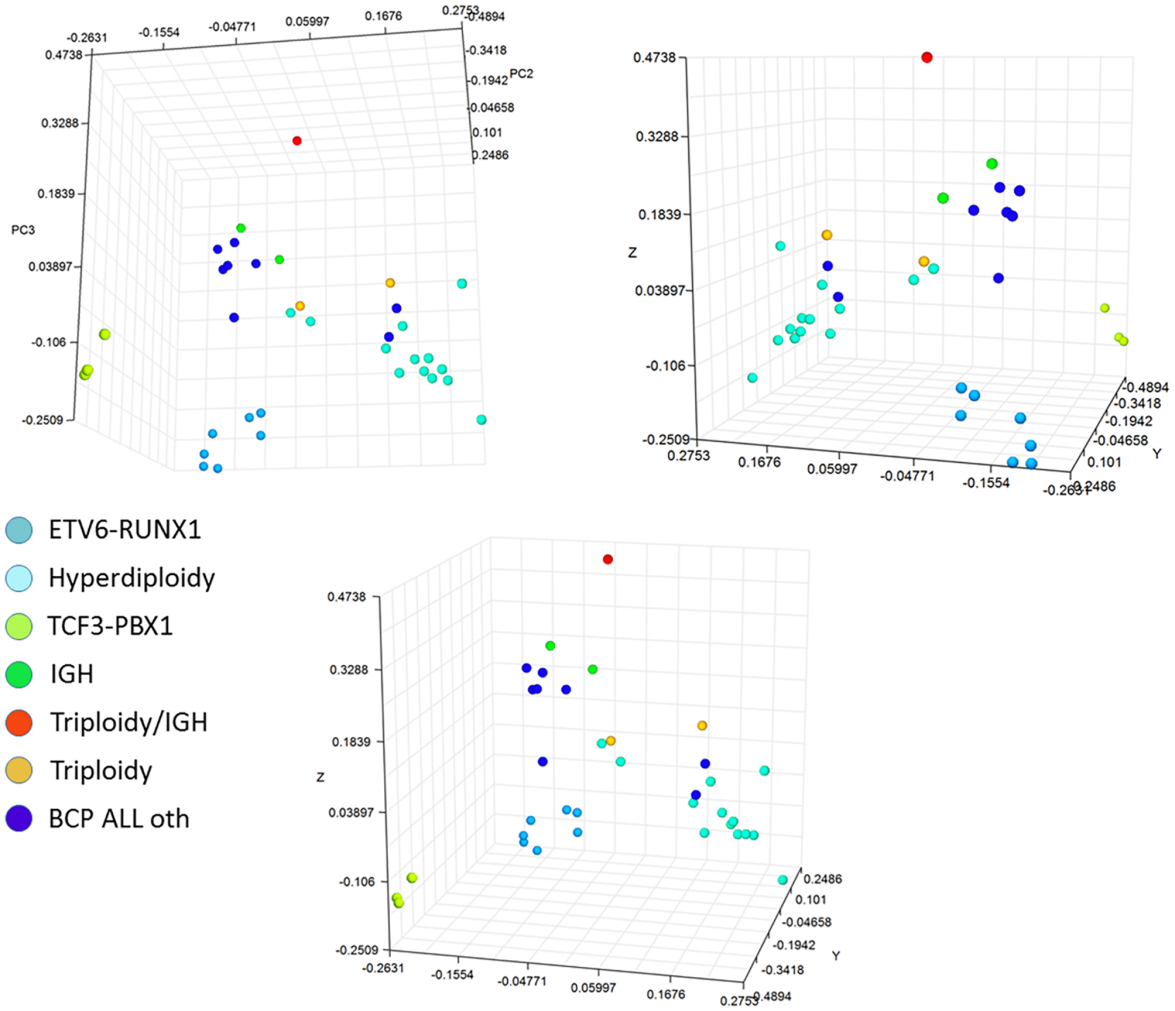
Principal component analysis 3D plot based on 500 probes with the highest differences in methylation level among genetic subtypes and remaining pre-B ALL patients. The plot is presented in three different layouts to enable visualization of separate clusters.

To determine the abilities of the sites that differed between separate genetic subtypes, from each comparison the top 100 DM sites (with the lowest adjusted p-value) were selected and a common panel of 500 sites was created with potential differentiation and diagnostic abilities. Unsupervised hierarchical clustering of the methylation level of selected probes allowed visible separation *of ETV6-RUNX1* and *TCF3-PBX1* profiles and classification of the remaining subtypes into mixed clusters with clear homogeneity (Fig 9). The three dimensional PCA plot allowed to clearly distinguish genetic leukemia subtypes associated with *ETV6-RUNX1* and *TCF3-PBX1* variants with visible separation of clusters including hyperdiploidy cases and the separation of a single case with IGH/triploidy aberrations (Fig 10). The list of probes used to distinguish the genetic BCP ALL subtypes are presented in S5 Appendix.

**Fig 11:**
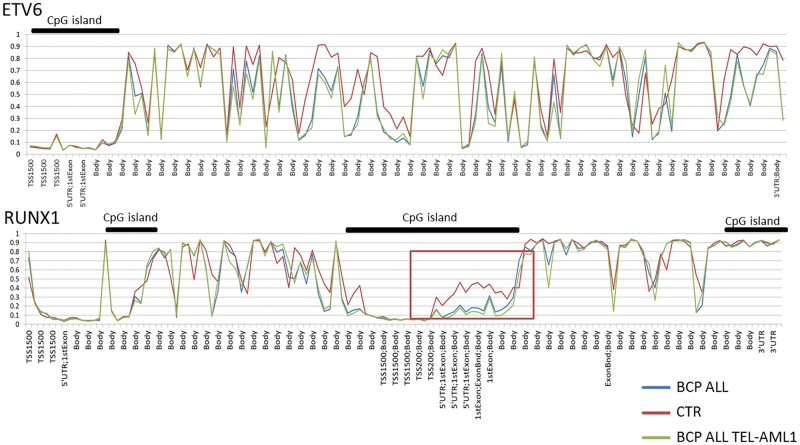
Methylation profile of *ETV6* and *RUNX1* genes in *ETV6-RUNX1* subtype patients, remaining pre-B ALL cases and control individuals. Red square marks the CpG fragments with large differences in methylation level between pre-B ALL and control samples.

An additional analysis was performed to explore the methylation profile of genes that are substrates of the most common chromosomal translocations found in the studied BCP ALL cases. The methylation profile of *ETV6* and *RUNX1* (*TEL-AML1*) genes was found to be variable across gene bodies, but showed visible demethylation within CpG islands located in promoter regions (both in BCP ALL and control patient groups) (Fig 11). Several sites across the *ETV6* and *RUNX1* genes differ significantly at a pointwise level between BCP ALL and the control group. Few of these however, differ between BCP ALL patients and BCP ALL cases with *ETV6-RUNX1* aberration. Within the *RUNX1* gene, demethylation was observed within two alternative gene promoters with clear hypermethylation of the gene body. The most pronounced differences in methylation level between BCP ALL and the control group were observed within the CpG island located in the central part of the *RUNX1* gene, which is an alternative gene promoter (P2). This region, however, did not show noticeable differences in methylation level between BCP ALL and BCP ALL-*ETV6-RUNX1* carriers.

**Fig 12:**
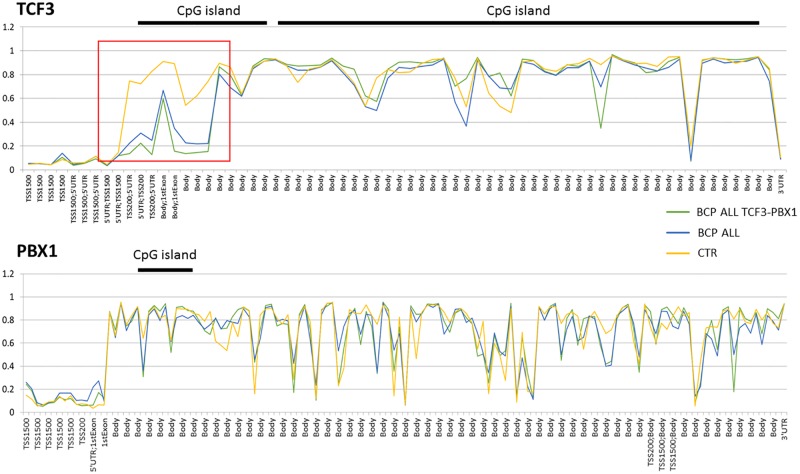
Methylation profile of *TCF3* and *PBX1* genes in *TCF3-PBX1* subtype patients, remaining pre-B ALL cases and control individuals. Red square marks the CpG fragments with large differences in methylation level between pre-B ALL and control samples.

A similar methylation pattern (demethylation of promoters and visible hypermethylation of gene bodies) was found for the *TCF3* and *PBX1* (*E2A-PBX1*) genes involved in t(1;19)(q23;p13) chromosomal rearrangement. The methylation profile of the genes was similar between BCP ALL, control and *TCF3-PBX1* rearrangement carriers, except the north-shore part of the CpG island located near the transcription start site of the *TCF3* gene shows hypermethylation in control group with respect to the BCP ALL patients (genome-wide adjusted p<0.05) (Fig 12).

It was possible using the lasso penalized logistic regression analysis to extract some DM sites related significantly with subsets of patients with *ETV6-RUNX1* and hyperdiploidy (Fig 7b and 7c). Among these sites, some were localized with promoter regions or gene bodies. The most important functional DM sites in particular subgroups are summarized in Table 5. These DM sites can potentially distinguish the groups with analyzed chromosomal aberrations after being confirmed in future studies with a larger patient cohort. The other cytogenetic aberrations were not analyzed using this method due to an insufficient number of affected patients.

**Table 5:** The most important DM sites in patients from particular BCP ALL subgroups extracted using the lasso penalized logistic regression analysis.

BCP ALL subgroup	Number of significant DM sites	Involved genes	Coefficients	Localization	Methylation status	potential function*
High risk group according ALL IC-BFM 2009	7	*MBP*	-8.13	ch.18 / gene body	Hypo methylated	- MBP transcription unit is an integral part of the Golli transcription unit and that this arrangement is important for the function and/or regulation of these genes
						- MBP-related transcripts are also present in the bone marrow and the immune system
		*PSMF1*	-7.96	ch.20/ TSS1500	Hypo methylated	- plays an important role in control of proteasome function.
						- the processing of class I MHC peptides.
						- Among its related pathways are RET signaling and Regulation of activated PAK-2p34 by proteasome mediated degradation.
Hyperdiploidy	11	*SYMPK*	23.16	ch.19 / gene body	Hyper methylated	- the regulation of polyadenylation and promotes gene expression
						- participates in 3'-end maturation of histone mRNAs
		*LINC00544*	-3.96	ch.13/ gene body	Hypo methylated	- long Intergenic Non-Protein Coding RNA
		*ATP11B*	-10.73	ch.3/ gene body	Hypo methylated	- nucleotide binding *and* cation-transporting ATPase activity.
		*MTHFD2L*	-10.56	ch.4/ gene body	Hypo methylated	- metabolism and metabolism of water-soluble vitamins and cofactors.
						- formate-tetrahydrofolate ligase activity *and* methylenetetrahydrofolate dehydrogenase (NADP+) activity.
		*BANF1*	12.80	ch.11/ TSS 1500	Hyper methylated	- plays fundamental roles in nuclear assembly, chromatin organization, gene expression and gonad development.
						- may potently compress chromatin structure and be involved in membrane recruitment and chromatin decondensation during nuclear assembly
		*C4orf51*	140.16	ch.4/ gene body	Hyper methylated	- uncharacterized protein
t(12;21) *TEL/AML1*	4	*SNTB1*	3.93	ch.8/3’UTR	Hyper methylated	- binding to and probably organizing the subcellular localization of a variety of membrane proteins.
		*GRHL3*	21.86	ch.1/gene body	Hyper methylated	- transcription factor activity
						- sequence-specific DNA binding *and* RNA polymerase II transcription factor activity
						- sequence-specific DNA binding
		*cTTN*	33.44	ch.11/ TSS200	Hyper methylated	- overexpressed in breast cancer and squamous cell carcinomas of the head and neck
						- regulating the interactions between components of adherens-type junctions
						- organizing the cytoskeleton and cell adhesion structures of epithelia and carcinoma cells
						- during apoptosis, the encoded protein is degraded in a caspase-dependent manner
						- the aberrant regulation of this gene contributes to tumor cell invasion and metastasis.
		*SLCO3A1*	4.86	ch.15/	hyper	- transport of glucose and other sugars, bile salts and organic acids, metal ions and amine compounds
				TSS1500	methylated	
						- transport of vitamins, nucleosides, and -related molecules

* according to http://www.genecards.org accessed 2017/05/04

## Discussion

### A distinct DNA methylation pattern of leukemic samples

The normal hematopoietic tissue in bone marrow consists of a variety of cell types including the blood cells and their precursors at different developmental stages derived from pluripotential, self-renewing stem cells and the microenvironment’s cells like adventitial reticular cells, endothelial cells, macrophages, adipocytes, bone lining cells (e.g., osteoblasts). During blood-cell development, pluripotent stem cells undergo either self-renewal or differentiation into multilineage committed progenitor cells: myeloid stem cells which can then differentiate into erythroid and myeloid (including granulocytes, megakaryocytes and macrophages) or lymphoid cell line. The neoplastic transformation of early lymphoblastic progenitor cells in BCP ALL dramatically changes their phenotype resulting in rapid proliferation and expansion. These abnormal lymphoblasts quickly dominate in bone marrow cavities; comprising up to 100% of the bone marrow cells. Due to this, the population of bone marrow cells obtained from patients with BCP ALL is practically homogenous compared to normal samples, where many cells of different origin and of different developmental stage occur. In this second, instance the poorly differentiated lymphocytic and myelocytic progenitors consist of up to 5% bone marrow cellularity. Consequently, the differences of methylation profiles between BCP ALL and normal bone marrow controls observed here-in can be explained by the heterogeneity of cells types in normal bone marrow, epigenetic control of the natural progenitor’s development and with methylation changes dependent with neoplastic transformation in leukemic samples.

The clear separation of DNA methylation profile between leukemic and normal bone marrow cells agrees with previous results [26–31]. Abnormal DNA methylation has been confirmed in many cancers including hematological neoplasms [32,33]. The role of DNA methylation in hematopoietic regulation has also been established [34]. For example, DNA methylation within bone marrow stem cells is important for their self-renewal and methylation deficiency leads to their differentiation into myeloerythroid progenitors [35]. Epigenetic control also plays a key role in lymphoid B-cells development as shown by Almamun *et al*. [36].

The differences in DNA methylation pattern between leukemic and normal bone marrow samples in our groups arise from many sites– 118,871 probes mapped to 17,885 unique genes or intergenic regions. Among them, a slight domination of hypermethylated over hypomethylated *loci* (56:44) in BCP ALL samples was revealed. Moreover, these DM sites are more commonly associated with CpG islands and in close vicinity of transcription start sites. Interestingly, the promoter-associated sites with different methylation were mainly (in 86,6% cases) hypermethylated in leukemia when compared to healthy controls. This may result in altered expression of many genes. The prevalence of differential hypermethylated promoter-associated CpG islands compare to non-diseased samples was detected also in other studies [26,28,30].

### Functional analysis of differential methylated genes (DMGs)

CpGs with distinct leukemic methylation were associated with 184 different genes (mainly gene bodies). Those genes are enriched in several GO biological processes, of which the most interesting appears to be: leukocyte activation (14 genes; adjP = 0.0097) or immune system process (32 genes; adjP = 0.0068). The genes were also over-represented in six different KEGG pathways, including the “pathways in cancer” term (6 genes; adjP = 0.0043). When the WikiPathways terms were interrogated, the most enriched was associated with the integrated pancreatic cancer pathway (5 genes; adjP = 0.0011).

The most meaningful results were however obtained, when terms from the PharmGKB database of disease phenotypes were analyzed. Several consistent disease phenotypes have been enriched by the analyzed genes, including: lymphoma, B-cell lymphoma, lymphoproliferative disorders, lymphatic diseases, lymphoid leukemia and leukemia (Table 6; S6 Appendix).

**Table 6:** Selected disease phenotypes enriched by genes associated with uniform leukemia methylation pattern.

Disease	Disease ID	adjP	Genes
Lymphoma	DB_ID:PA444840	1.06E-05	*CXCR5*, *NCOR2*, *TNFAIP3*, *IL7R*, *TRAF2*, *RUNX1*, *CD27*, *FOXP1*, *EML4*, *MME*, *NBN*
Lymphoma, B-Cell	DB_ID:PA446304	1.60E-05	*CXCR5*, *NCOR2*, *TNFAIP3*, *IL7R*, *TRAF2*, *RUNX1*, *CD27*, *FOXP1*, *MME*
Cancer or viral infections	DB_ID:PA128407012	0.0002	*MX1*, *TIMP2*, *HIF1A*, *ERCC3*, *NDRG1*, *FANCD2*, *RUNX1*, *BLM*, *FOXP1*, *EML4*, *MME*, *IFITM1*, *SMARCB1*, *NBN*
Lymphoma, Low-Grade	DB_ID:PA446307	0.0002	*CXCR5*, *TNFAIP3*, *CD27*, *FOXP1*, *TRAF2*, *MME*, *NBN*
Lymphoma, B-Cell, Marginal Zone	DB_ID:PA446727	0.0002	*TNFAIP3*, *CD27*, *FOXP1*, *TRAF2*
Lymphoproliferative Disorders	DB_ID:PA444849	0.0002	*CXCR5*, *TNFAIP3*, *IL7R*, *TRAF2*, *RUNX1*, *CD27*, *FOXP1*, *MME*, *NBN*
Lymphatic Diseases	DB_ID:PA444833	0.0002	*CXCR5*, *TNFAIP3*, *IL7R*, *TRAF2*, *RUNX1*, *CD27*, *FOXP1*, *MME*, *NBN*
Lymphoma, Non-Hodgkin	DB_ID:PA444845	0.0004	*CXCR5*, *TNFAIP3*, *CD27*, *BLM*, *TRAF2*, *NBN*
Virus Diseases	DB_ID:PA446038	0.0006	*MX1*, *CXCR5*, *ITGAL*, *IL7R*, *TANK*, *PSMB7*, *PIK3CD*, *CD27*, *IFITM1*
Leukemia, Lymphoid	DB_ID:PA444756	0.0006	*CXCR5*, *RUNX1*, *BRE*, *CD27*, *IL7R*, *MME*, *LINC00598*
Lymphoid leukemia NOS	DB_ID:PA165108377	0.0006	*CXCR5*, *RUNX1*, *BRE*, *CD27*, *IL7R*, *MME*, *LINC00598*
Precursor Cell Lymphoblastic Leukemia-Lymphoma	DB_ID:PA446155	0.001	*RUNX1*, *IL7R*, *MME*, *LINC00598*, *NBN*
Leukemia	DB_ID:PA444750	0.0018	*CXCR5*, *NCOR2*, *RUNX1*, *BLM*, *CD27*, *SENP1*, *MME*, *NBN*

When 124 genes related with hypermethylated CpGs were screened separately for their functions, only a single KEGG pathway was found to be significant and is related to protein processing in endoplasmic reticulum (4 genes; adjP = 0.0030). The hypermethylated genes were also enriched in several disease phenotypes with one which could be directly connected with cancer, namely: hepatocellular carcinoma phenotype (4 genes; adjP = 0.0075).

When the remaining 60 genes associated with hypomethylated sites were analyzed separately, no enriched biological processes, molecular functions or pathways were found. However, all previously mentioned lymphoma/leukemia associated disease phenotypes were enriched by this gene category.

The analyzed promoter regions were associated with 2045 different genes. The annotation analysis showed, that hypermethylated probes were found within putative promoter regions of 1540 genes and hypomethylated sites were associated with 518 genes. When the genes were analyzed jointly in terms of their functional relevance, several general biological processes or processes connected with nervous system functioning and development have been detected, including e.g.: nervous system development, single-multicellular organism process, neuron differentiation, neurogenesis, cell-cell signaling and many others. Among the top ten significantly enriched KEGG pathways there were those associated, for example, with neuroactive ligand-receptor interaction (68 genes; adjP = 4.21e-30), calcium signaling (36 genes; adjP = 6.73e-13) or pathways in cancer (34 genes; adjP = 6.29e-05). Among the top ten enriched disease phenotypes, no leukemia-related disorders were found. When genes with hyper- and hypomethylated promoters were analyzed separately, however, we find that the hypermethylated genes are enriched in disease phenotypes associated with nervous system and mental disorders, and that the hypomethylated genes are enriched several disease phenotypes, like e.g.: lymphoproliferative disorders, lymphoid leukemia, B-Cell lymphoma, Lymphoid leukemia NOS, lymphatic diseases, general leukemia or B-Cell leukemia (Table 6 and S7 Appendix).

The results of other studies have revealed that the spectrum of genes with potentially aberrated methylation in BCP ALL is very wide and includes many biological processes. These processes include, among others, growth and proliferation’s regulation [26,29–31], apoptosis [26,29,30,37] hematopoiesis and lymphocyte B development [27,28,36] and immune response [38]. It must be noted that alteration of promoter DNA methylation does not have to translate to major changes in gene expression in any case [26].

### The impact of methylation signature on prognosis in BCP ALL

The discovery of new molecular prognostic factors is important for further therapy individualization and optimization. The meaningful differences in genes methylation between HR and IR/SR BCP ALL patients were depended with both: promoters as well as gene bodies regions. Promoter region-related sites were connected with *PSMF1* and *DIP2B* genes, whereas differential methylation of CpGs within gene bodies was found in *NGFR*, *ADAMTS20* and *MBP* genes. Although there was not enough data for conclusive results we also tried to extract the differences between methylation of single/multiple genes in patients stratified by risk group. We observe only minimal differences between count of DM CpG sites in HRG and *IRG/SRG* patients but it was possible to distinguish seven DM sites which could be the biomarkers of the HR group. The risk prediction based on these 7 DM sites might be determined early, before therapy response assessment. Two of these 7 sites, are localized in gene body/promoter regions, and thus may have potential functional roles in regulating the expression of *MBP* and *PSMF1* genes. [Table 5]. The protein encoded by the classic *MBP* gene is a major constituent of the myelin sheath of oligodendrocytes and Schwann cells in the nervous system. However, *MBP*-related transcripts are also present in the bone marrow and the immune system [39]. The *MBP* has also an important role to play in apoptosis [40]. It has been shown that *MBP* SNP rs3794845 is significantly associated with childhood ALL risk [41]. *PSMF1* gene encodes a protein that inhibits the activation of the proteasome by the 11S and 19S regulators (provided by apoptotic and cell cycle mechanisms). Proteasome inhibitors (for example bortezomib) enhance many conventional therapies, also in ALL [42]. Although there are some limitations including small sample size and a lack of genes’ expression information supporting the aberrated DM sites methylation, our study identified two genes with potential impact on childhood ALL prognosis.

Global promoter methylation profiling has been previously demonstrated to all subgrouping and prognostication of pediatric ALL in T cells phenotype ALL [43,44]. Takeuchi *et al* [45] revealed that patients from medium/high risk group had multiple genes methylated compared to those from low risk but they included patients with both, B- and T-cells ALL together. No specific methylation profile characteristic for *HRG* was observed in our study. The relationship between methylation and outcome prognosis or relapse risk in pediatric BCP ALL is unproven. Some authors [28,30,46] have identified some genes with aberrant methylation relevant to relapse prediction but others [38] reached opposing conclusions and didn’t find any statistically significant associations between methylation level of any CpGs and subsequent relapse of BCP ALL.

### Epigenetic landscape and cytogenetic subtypes in childhood BCP ALL

Our study confirmed the distinguishable differences in the general methylation pattern between the analyzed cytogenetic subsets of patients. It was possible to clearly separate genetic leukemia subtypes associated with *ETV6-RUNX1* and *TCF3-PBX1* variants with visible separation of clusters including hyperdiploidy cases and separation of the single case with IGH/triploidy aberrations. These findings are consistent with observations from other studies [30,38,47] where the cytogenetic subtypes showed a clear correlation with methylation profile but this clustering was not absolute.

The lack of clear differences in methylation level of genes involved in chromosomal translocations between BCP ALL patients with- or without specific rearrangement, suggest that the translocation and change of genes’ sequence context does not affect methylation and that methylation seems not to be a mechanism for the regulation of expression of the resulting fusion genes. The methylation profile of some of these genes, however, show signs of alteration between healthy and leukemia samples.

Methylation sites with potential significant different methylation status specifically depended on analyzed cytogenetic status. Only a few of these sites are in gene bodies or promoter regions. Their functional role stays unknown but they may be potentially used as biomarker of ALL patients with hyperdiploidy or t(12;21) and should be confirmed in future studies with a larger cohort of patients. Genes specifically correlated with analyzed cytogenetic subsets (listed in Table 5) may be suitable targets for the searching new individualized BCP ALL therapy.

## Conclusions

The clear separation of DNA methylation profile between leukemic and normal bone marrow cells was confirmed. The analyzed translocations and change of genes’ sequence context does not affect methylation thus methylation seems not to be a mechanism for the regulation of expression of the resulting fusion genes. Moreover, it was possible to distinguish some DM sites which could be the biomarkers of the BFM ALL IC 2009 high risk group as well as hyperdiploidy and t(12;21) ETV6-RUNX1 carriers.

## Supporting information

S1 Appendix A set of 118,871 probes differentially methylated (adjP<0.05) in all ALL_B patients relative to controls. (XLSX)

S2 Appendix CpG sites with low variation among ALL_B group and differentially methylated with respect to control. (XLSX)

S3 Appendix A list of CpG sites differentially methylated between *HRG* and *SRG/IRG* leukemia patients. (XLSX)

S4 Appendix The list of probes differentially methylated between genetic subtypes and remaining BCP ALL patients, along with their annotation and associated genes. (XLSX)

S5 Appendix The list of 500 probes with the highest differences in methylation level among genetic subtypes and remaining BCP ALL patients. (XLSX)

S6 Appendix Disease phenotypes enriched by genes associated with uniform CpG methylation profile in leukemia patients. (XLSX)

S7 Appendix Disease phenotypes enriched by genes with hypomethylated CpG sites in leukemia samples across genome (promoters and gene bodies). (XLSX)
